# HIV Latency Is Established Directly and Early in Both Resting and Activated Primary CD4 T Cells

**DOI:** 10.1371/journal.ppat.1004955

**Published:** 2015-06-11

**Authors:** Leonard Chavez, Vincenzo Calvanese, Eric Verdin

**Affiliations:** 1 Gladstone Institute of Virology and Immunology, San Francisco, California, United States of America; 2 Biomedical Sciences Program, University of California, San Francisco, San Francisco, California, United States of America; 3 Department of Medicine, University of California, San Francisco, San Francisco, California, United States of America; Fred Hutchinson Cancer Research Center, UNITED STATES

## Abstract

Highly active antiretroviral therapy (HAART) suppresses human immunodeficiency virus (HIV) replication to undetectable levels but cannot fully eradicate the virus because a small reservoir of CD4^+^ T cells remains latently infected. Since HIV efficiently infects only activated CD4^+^ T cells and since latent HIV primarily resides in resting CD4^+^ T cells, it is generally assumed that latency is established when a productively infected cell recycles to a resting state, trapping the virus in a latent state. In this study, we use a dual reporter virus—HIV Duo-Fluo I, which identifies latently infected cells immediately after infection—to investigate how T cell activation affects the estab-lishment of HIV latency. We show that HIV latency can arise from the direct infection of both resting *and* activated CD4^+^ T cells. Importantly, returning productively infected cells to a resting state is not associated with a significant silencing of the integrated HIV. We further show that resting CD4^+^ T cells from human lymphoid tissue (tonsil, spleen) show increased latency after infection when compared to peripheral blood. Our findings raise significant questions regarding the most commonly accepted model for the establishment of latent HIV and suggest that infection of both resting and activated primary CD4^+^ T cells produce latency.

## Author Summary

The study of HIV latency has been hindered because there are few latently infected cells *in vivo*, and we cannot distinguish latently infected cells from uninfected cells prior to reactivation of the latent provirus. In general, HIV latency is quantitatively studied by reactivating latently infected cells after latency has been established. However, this practice limits the investigation of how latency is established and how latent provirus can be reactivated. Our recently developed dual reporter virus, HIV Duo-Fluo I, can identify latently infected cells early after infection. In this study, we use HIV Duo-Fluo I to investigate how T cell activation affects the outcome of HIV infection.

## Introduction

Once highly active antiretroviral therapy (HAART) became available in 1995, HIV infection was transformed from a deadly disease into a chronic lifelong condition [1]. The antiretroviral drugs used in HAART target multiple stages of the viral lifecycle, which can reduce patient viremia to undetectable levels [2–4]. However, HAART cannot eradicate HIV [5] because infected individuals harbor a small reservoir of latently infected cells that contain a transcriptionally silent but reactivatable provirus [6]. Because this latent reservoir prevents viral eradication, there is an urgent need to study and better understand the mechanisms of latency.

HIV infection primarily targets CD4^+^ T cells, and the most extensively studied latent reservoir resides within resting CD4^+^ T cells [7–9]. During infection, HIV enters a target cell and reverse-transcribes its genomic viral RNA into a double-stranded cDNA that then enters the nucleus and integrates into the host genome, where it becomes controlled by the host transcriptional machinery. In most cases, integration of the viral genome leads to productive infection, in which viral genes are transcribed followed by virion production. However, in rare instances, latency occurs instead of productive infection and is characterized by a provirus that produces little-to-no viral transcripts [10]. Because the latently infected cell is not producing viral proteins, it escapes the viral cytopathic effects and is ignored by the immune system. Furthermore, since antiretroviral drugs only target active viral replication, they are ineffective against latent proviruses. Latent HIV is primarily found within memory CD4^+^ T cells, which have a long half-life *in vivo* [11, 12], allowing latent virus to persist within infected individuals for decades [13]. However, when latently infected memory CD4^+^ T cells encounter an antigen or are exposed to specific cytokines or chemokines, proviral transcription is activated, leading to productive infection [8, 14]. This “reactivation” is likely the cause of viral rebound after a patient stops HAART, and it explains why infected individuals must take antiretroviral drugs for life.

HIV latency has proven difficult to study because latently infected cells are very rare *in vivo* (~1 in 1 × 10^6^ cells) [11], and they cannot be distinguished from uninfected cells [15]. Despite these challenges, several *in vitro* latency models exist, which have led to important observations about how latently infected cells are maintained and reactivated (reviewed in references [16, 17]).

**Fig 1:**
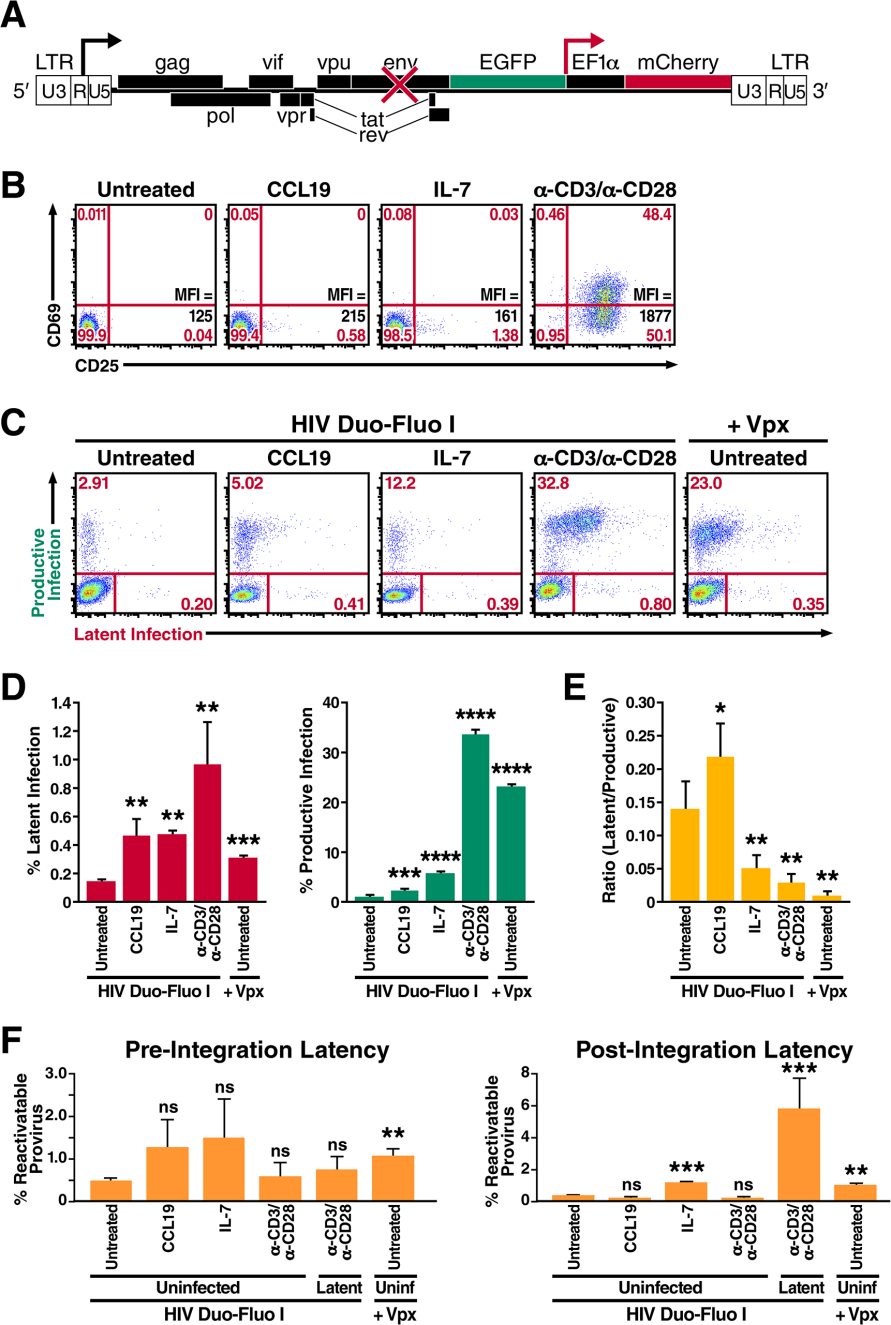
Resting primary CD4^+^ T cells support both productive and latent HIV infection. (A) Diagram of the HIV Duo-Fluo I virus, in which eGFP has replaced the *nef* gene, and a whole transcription unit—consisting of an EF1α promoter driving the expression of an mCherry fluorescent marker—has been inserted downstream. Upon infection with the HIV Duo-Fluo I virus, cells that express GFP alone or GFP and mCherry are considered productively infected; cells that express only mCherry are considered latently infected; cells that lack expression of either fluorescent marker are considered uninfected. (B) Expression of the activation markers CD69 and CD25 in resting primary CD4^**+**^ T cells either left untreated or stimulated with CCL19, IL-7, or αCD3/αCD28 activating beads for 72 h. Mean Fluorescence Intensity (MFI) for CD25 expression is also shown. (C) Infection profiles of untreated or stimulated primary CD4^**+**^ T cells 6 days after infection via flow cytometry. Untreated resting CD4^**+**^ T cells were infected with either HIV Duo-Fluo I virus alone or the Vpx-containing HIV Duo-Fluo I virus. Stimulated cells were infected with HIV Duo-Fluo I alone. Productive infection (GFP+ and GFP/mCherry double-positive) and latent infection (mCherry+) were analyzed by flow cytometry. Data shown are from a single donor but are representative of three separate donors. (D) Quantified values of latent infection and productive infection from panel C. Data represents the average of three donors. (E) Ratios of latent infection to productive infection were calculated using data from panel D. Data represent the average of three donors. (F) Quantified values for reactivation of pre-integration latent virus and post-integration provirus calculated from the isolated uninfected populations (GFP/mCherry double-negative) of untreated and stimulated primary CD4^**+**^ T cells via flow cytometry (S4 Fig). Six days after infection, uninfected cells were isolated via fluorescence-activated cell sorting (FACS) and were either left unstimulated or stimulated with αCD3/αCD28 activating beads alone or αCD3/αCD28 activating beads in the presence of raltegravir for 48 h. Reactivatable pre-integration latent virus was calculated by subtracting the amount of productive infection from cells treated with αCD3/αCD28 activating beads alone and cells treated with αCD3/αCD28 activating beads in the presence of raltegravir. Reactivatable post-integration latent provirus was calculated by subtracting the amount of productive infection from unstimulated cells and cells treated with αCD3/αCD28 activating beads in the presence of raltegravir. Data represent the average of three donors. *, P < 0.05; **, P < 0.01; ***, P < 0.001; ****, P < 0.0001; ns, non-significant.

However, it is not clear how the latent reservoir is established because current technologies only quantify latently infected cells by reactivating them from latency. We recently developed a dual reporter virus, HIV Duo-Fluo I, that can distinguish between cells that are productively infected, latently infected, or uninfected, and allows us to purify each population [18]. Using this new reporter virus, we can study the kinetics of HIV latency immediately after infection by employing two separate fluorescent markers: an LTR-driven eGFP marker (productive infection) and an LTR-independent mCherry marker (latent infection) driven by an EF1α promoter (Fig 1A). It should be noted that we use the term “productive infection” here and throughout the manuscript to indicate an infection resulting in the expression of the LTR-driven GFP reporter. Since the virus used in this manuscript is env-deficient, these infections are not truly productive. However, they are behaving like a productive infection in terms of virus expression levels. Using this dual reporter virus, we have studied how HIV latency is established with a unique focus on the role of T cell activation.

Based primarily on *in vitro* evidence, it is generally accepted that HIV predominantly replicates in activated CD4^+^ T cells [19–22]. Conversely, resting CD4^+^ T cells present several barriers to HIV infection (reviewed in reference [23]), as they do not support efficient nuclear import [24] or integration of the viral cDNA [22, 25]. However, the most notable obstacle to infection of resting CD4^+^ T cells occurs at the stage of reverse transcription [26, 27]. Resting CD4^+^ T cells do not support reverse transcription nearly as efficiently as activated cells because, at least in part, they contain the restriction factor SAMHD1 [28, 29]. Additionally, *in vivo*, most HIV-infected resting CD4^+^ T cells exhibit a memory phenotype, suggesting that they arose from the infection of previously activated CD4^+^ T cells. Based on this evidence, a leading theory postulates that latency is established from infected activated CD4^+^ T cells that revert back to a resting memory state. According to this model, the transition to a resting memory state is associated with a decrease in NFκB and pTEFb activity, two critical factors for HIV transcription, and with a concomitant silencing of the HIV genome [30]. However, for this type of latency to occur, the infected cell would have to survive the virus-induced cytopathic effects and the host immune response that usually kill productively infected cells very quickly (cells survive ~1 day) [31, 32]. Another possibility is that infection occurs at a “sweet spot” in the trajectory that activated CD4^+^ T cells taken from full activation to a fully rested state. This sweet spot would be characterized by permissivity for HIV reverse transcription and integration but not for HIV transcription [33].

Interestingly, previous studies have reported that resting CD4^+^ T cells can be directly infected, with the strongest evidence coming from *in vivo* and *ex vivo* studies of both SIV and HIV infection [34–39]. Most studies that show resting CD4^+^ T cells can be directly infected have been performed with cells isolated from primary lymphoid tissues. *In vivo* studies have found that resting CD4^+^ T cells in lymphoid tissue harbor viral RNA [35], and *ex vivo* studies have shown that directly infecting resting CD4^+^ T cells from lymphoid tissue results in productive infection [40]. Strikingly, a subsequent study found that resting CD4^+^ T cells in *ex vivo* lymphoid cells isolated from tonsillar tissue can support HIV infection, but purified CD4^+^ T cells isolated from that same lymphoid tissue could not [41], suggesting that the lymphoid tissue microenvironment is critical for rendering resting CD4^+^ T cells permissive to HIV infection. Indeed, several lymphoid tissue–associated factors, including cytokines [42], chemokines [43], extracellular matrixes [44], and cell surface markers [45], enhance HIV infection in resting CD4^+^ T cells. Therefore, HIV latency may be established by the direct infection of resting CD4^+^ T cells when they are exposed to soluble factors that do not induce classic T cell activation.

In this study, we use the dual reporter virus, HIV Duo-Fluo I, to investigate the role of T cell activation on the establishment of HIV latency in primary CD4^+^ T cells. We also use HIV Duo-Fluo I to explore the theories of how HIV latency is established; namely, whether it occurs through infection of activated CD4^+^ T cells that return to a resting state or through the direct infection of resting CD4^+^ T cells. We find that both resting and activated primary CD4^+^ T cells can support both productive and latent infection. In the case of activated T cells, the latent state is established very early in the infection and is not significantly influenced by the return of that activated cell to a resting state. We further observed that the fraction of cells that become latent (latent/productive) is higher in resting CD4^+^ T cells than in activated CD4^+^ T cells.

## Results

### Resting primary CD4^+^ T cells support both productive and latent infection but are biased toward latent infection

The literature is replete with conflicting reports on whether resting CD4^+^ T cells can be infected by HIV, either productively or latently [23]. Many studies indicate that resting CD4^+^ T cells are refractory to productive HIV infection but can become permissive to infection after treatment with certain cytokines or chemokines that do not induce classic T cell activation [46, 47]. To test the permissivity of resting CD4^+^ T cells to our HIV Duo-Fluo I virus, we isolated total CD4^+^ T cells from peripheral blood of uninfected donors via depletion of all non-CD4^+^ T cells (negative selection). These cells did not express CD69 or CD25 (Fig 1B) and can therefore be considered resting. These cells were either left untreated or were treated with the cytokine IL-7 or the chemokine CCL19 for 72 h prior to infection. Stimulation with IL-7 or CCL19 slightly increased CD25 expression, such that 0.58% and 1.38% of cells expressed CD25, respectively (Fig 1B). As a positive control, resting CD4^+^ T cells were stimulated with αCD3/αCD28 activating beads in the presence of IL-2 for 72 h prior to infection, which led to significant expression of both CD69 and CD25 activation markers (Fig 1B).

Both untreated and treated cells were spinoculated with HIV Duo-Fluo I for 2 h at 37°C and then returned to culture in the presence of IL-2. Productive infection (GFP+ and mCherry+/GFP+) and latent infection (GFP-/mCherry+) were monitored daily by flow cytometry for 6 days following infection (S1 Fig). Compared to αCD3/αCD28-stimulated cells at 6 days postinfection, untreated resting CD4^+^ T cells showed significantly lower levels of HIV infection but, nonetheless, permitted both productive and latent infection (Fig 1C). Importantly, productive and latent infection of resting CD4^+^ T cells over the 6-day time-course was not the result of replication-competent virus being present in our HIV Duo-Fluo I viral stocks (S2 Fig) [48].

Despite minimally affecting T cell activation, both IL-7 and CCL19 treatment led to an increase in HIV infection compared to untreated resting CD4^+^ T cells (Fig 1C and 1D), suggesting that the permissibility of resting CD4^+^ T cells can be enhanced without undergoing classic T cell activation, which agrees with previous studies [47, 49]. However, we show how productive and latent infection is distributed within resting CD4^+^ T cells after such treatments (Fig 1D). Treating resting CD4^+^ T cells with CCL19 increased productive infection 2-fold over untreated cells, while IL-7 treatment produced a 5-fold increase in productive infection. Latent infection increased by 3-fold after treatment with either CCL19 or IL-7.

In addition to cytokine and chemokine treatment, we also investigated the role of the human protein SAMHD1 in restricting HIV infection within resting CD4^+^ T cells. To do this, we infected resting CD4^+^ T cells with HIV Duo-Fluo I containing Vpx, which was provided in *trans* as a fusion protein with Vpr. Vpx is a lentiviral accessory protein encoded by HIV-2 that degrades SAMHD1 and thereby allows the virus to infect many cell types—resting CD4^+^ T cells [28], dendritic cells, monocytes, and macrophages [50, 51]—that are usually off limits to HIV-1 because of a SAMHD1-imposed post-entry block. Infecting untreated resting CD4^+^ T cells with Vpx-containing HIV Duo-Fluo I increased their infection levels over untreated cells infected with HIV Duo-Fluo I alone (Fig 1C and 1D). This increase in infection correlates with SAMHD1 protein down-regulation mediated by Vpx (S3A Fig) and has little to do with T cell activation (S3B Fig). As such, levels of productive infection increased significantly and were almost comparable to those of activated CD4^+^ T cells, whereas levels of latent infection were more than 2-fold greater than in resting untreated cells infected with HIV Duo-Fluo I alone. Overall, this increase in infection suggests that knocking down SAMHD1 in resting CD4^+^ T cells biases the cells toward productive infection, an observation that is confirmed by calculating the ratio of latently infected to productively infected cells (Fig 1E). Based on the ratio of latently infected to productively infected cells, IL-7–treated resting CD4^+^ T cells and activated CD4^+^ T cells also support more productive infection than latent infection. Conversely, untreated and CCL19-treated resting CD4^+^ T cells support more latent infection than productive infection.

To ensure that infecting resting CD4^+^ T with HIV Duo-Fluo I did not lead to any silent infection events—in which viral integration occurred but failed to produce expression of either fluorescent marker—we sorted the uninfected populations (GFP-/mCherry-) of both untreated and treated CD4^+^ T cells by FACS at 6 days post-infection. These cells were then stimulated with αCD3/αCD28 activating beads in the presence or absence of the integrase inhibitor, raltegravir, to distinguish between the reactivation of any pre-integration latent virus and post-integration latent provirus that might be present (S4 Fig). As a control, we isolated (via FACS) latently infected cells (GFP-/mCherry+) from infected cells that were pretreated with αCD3/αCD28 activating beads and subjected them to the same treatments as the uninfected cells. We analyzed reactivation of the latent virus by flow cytometry 48 h after stimulation. All cell populations contained some reactivatable pre-integration latent virus (Fig 1F); the highest levels were observed in CCL19- and IL-7-treated populations and in the untreated population infected with Vpx-containing HIV Duo-Fluo I. Untreated resting CD4^+^ T cells infected with the HIV Duo-Fluo I virus alone showed the lowest levels of reactivatable pre-integration latency, followed closely by activated CD4^+^ T cells.

By analyzing the reactivatable post-integration latent provirus, we found that the uninfected cell population isolated from activated CD4^+^ T cells contains very little reactivatable provirus (0.26%, Fig 1F) compared to the initial latent population identified in activated CD4^+^ T cells after infection (1.01%, Fig 1D). These findings suggest that HIV Duo-Fluo I can efficiently identify latently infected cells within activated CD4^+^ T cells. Conversely, uninfected cells isolated from untreated *resting* CD4^+^ T cells contained 0.40% reactivatable provirus, but only 0.14% latently infected cells were identified in this cell population after the initial infection. This finding suggests that HIV Duo-Fluo I identifies only a fraction of latently infected cells within resting CD4^+^ T cells. Similarly, IL-7–treated resting CD4^+^ T cells and untreated resting CD4^+^ T cells infected with the Vpx-containing HIV Duo-Fluo I both contained over 1% reactivatable provirus within their isolated uninfected cell populations, which was more than twice the size of the latent cell populations identified in these respective populations after the initial infection (Fig 1D). This suggests that IL-7 treatment and SAMHD1 knockdown lead to silent infection events in resting CD4^+^ T cells, and, thus, an underestimation of the latently infected cell population. Interestingly, CCL19 treatment of resting CD4^+^ T cells produced 0.2% reactivatable provirus from the isolated uninfected cell population, while the initial latent cell population after infection was 0.47%. Overall, these data suggest that HIV Duo-Fluo I can be used to identify latently infected cells within activated CD4^+^ T cells, but may underestimate the percentage of latently infected cells within resting CD4^+^ T cells.

### Primary CD4^+^ T cells become less permissive to HIV infection as they transition from an activated to a resting state but exhibit a higher propensity toward latent infection

**Fig 2:**
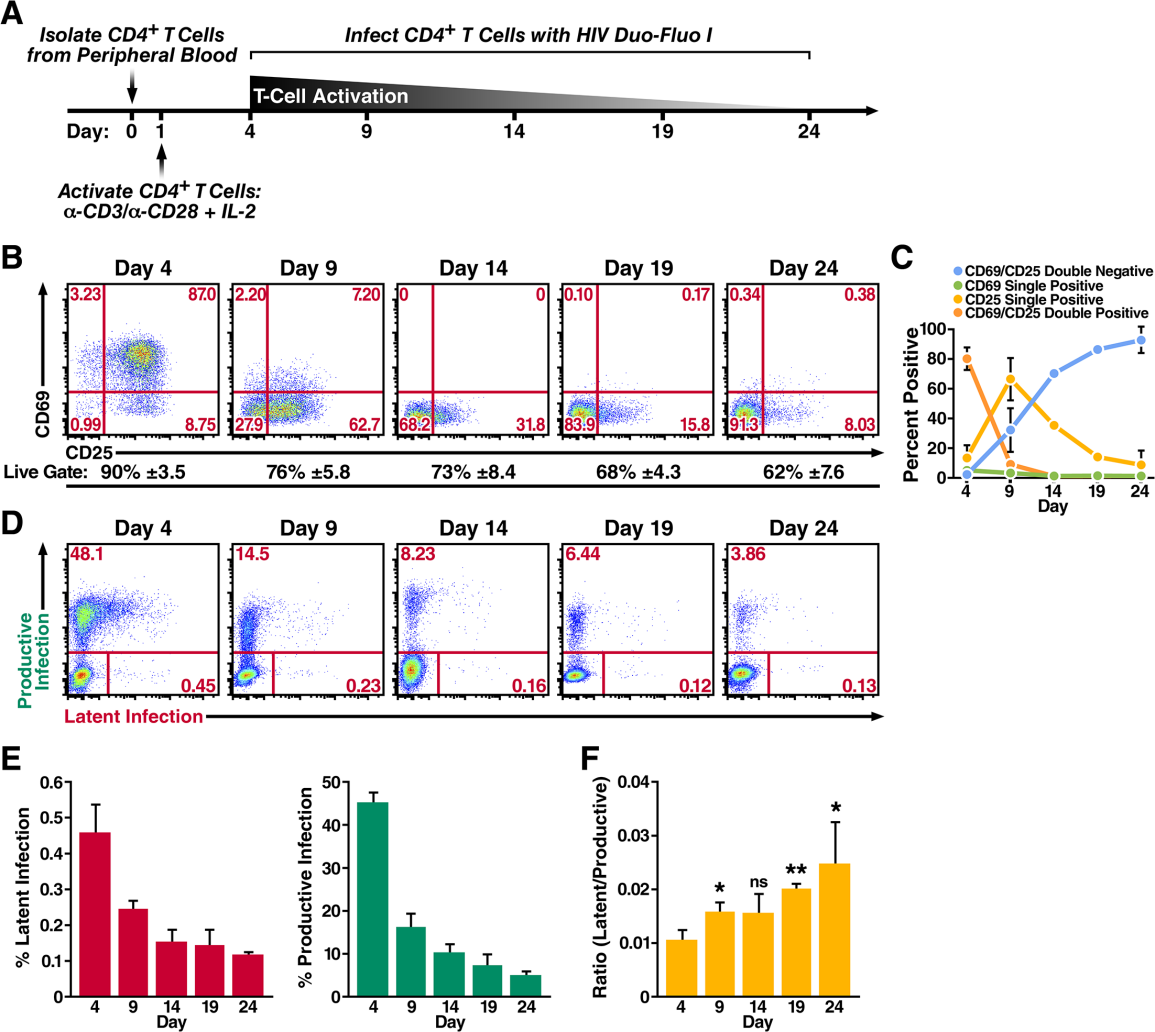
Primary CD4^+^ T cells transitioning from an activated state back to a resting state are more likely to become latently infected. (A) Schematic of experimental procedure. Primary CD4^**+**^ T cells were isolated from uninfected donor blood and stimulated with αCD3/αCD28 activating beads in the presence of IL-2 for 72 h and were then allowed to return to a resting state over 20 days in the presence of IL-2. Cells were infected at peak activation (day 4) and every 5 days thereafter as they returned to a resting state. (B) Expression of activation markers CD69 and CD25 as the cells transition from an activated state to a resting state. Flow cytometry was performed 72 h post activation and every 5 days after the activation beads were removed. Data shown are from a single donor, but representative of three separate donors. Percentage of live cells is calculated from the live gate (forward vs side scatter plots) in the FACS analysis and represents the average of three donors. (C) Quantified values of the cells’ activation status from panel B. Data represents the average of three donors. (D) Infection profiles of primary CD4^**+**^ T cells as they transition back to a resting state. Cells were spinoculated with HIV Duo-Fluo I 72 h after activation and every 5 days after the activation beads were removed. Infection was analyzed by flow cytometry 72 h post-infection. Data shown are from a single donor, but representative of three separate donors. (E) Quantified values of latent infection and productive infection from panel D. Data represents the average of three donors. (F) Ratios of latent infection to productive infection were calculated using data from panel E. Data represents the average of three donors. *, P < 0.05; **, P < 0.01; ns, non-significant.

Because HIV replicates most efficiently in activated CD4^+^ T cells [23], and the largest *in vivo* latent reservoir is within resting memory CD4^+^ T cells, we next investigated whether HIV latency is preferentially established in CD4^+^ T cells that become infected as they transition from an activated to a resting state. To do this, we isolated total CD4^+^ T cells from peripheral blood of uninfected donors and stimulated the cells with αCD3/αCD28 activating beads in the presence of IL-2 for 3 days (Fig 2A). We then removed the αCD3/αCD28 activating beads and allowed the cells to return to a resting state in the presence of IL-2 for 20 days. We infected the CD4^+^ T cells with HIV Duo-Fluo I at peak activation (day 4) and every 5 days thereafter as they transitioned back to resting. As indicated by expression of the activation markers CD69 and CD25, the cells transitioned from active to resting over the 20 days and remained >60% viable (Fig 2B). Maximal activation occurred at day 4 with 79% of cells expressing both CD69 and CD25 (Fig 2C). By day 9, however, 31% of cells no longer expressed CD69 or CD25, and by day 24, 92% of cells no longer expressed either activation marker. Despite most cells losing CD69 expression by day 14 (<1% CD69^+^), a small fraction of cells continued to express CD25 throughout the experiment—with 8% of cells still CD25^+^ at day 24—suggesting that while most CD4^+^ T cells had returned to a resting state by day 24, a small population was still transitioning back to a resting state. Others have observed similar expression profiles while trying to return activated CD4^+^ T cells to a resting state [52, 53].

We analyzed productive infection and latent infection by flow cytometry at 3 days postinfection for each time point (Fig 2D), and we determined the average of each cell population from three donors (Fig 2E). Infection at day 4, when the CD4^+^ T cells were maximally activated, produced the highest levels of both productive and latent infection. As the cells returned to a resting state, the levels of both productive infection and latent infection decreased, suggesting that HIV most effectively infects CD4^+^ T cells when they are at their highest activation state; as CD4^+^ T cells stop expressing the activation markers CD69 and CD25, they become less permissive. However, the ratio of latent infection to productive infection steadily increased from 0.010 at day 4 when the cells where most active to 0.025 at day 24 when the cells exhibited a more resting phenotype (Fig 2F). Therefore, while activated CD4^+^ T cells support the most robust infection, latent infection is more likely to occur relative to productive infection in cells that are resting or are transitioning back to a resting state.

### Productively and latently infected activated primary CD4^+^ T cells lose expression of both fluorescent markers as they return to a resting state

**Fig 3:**
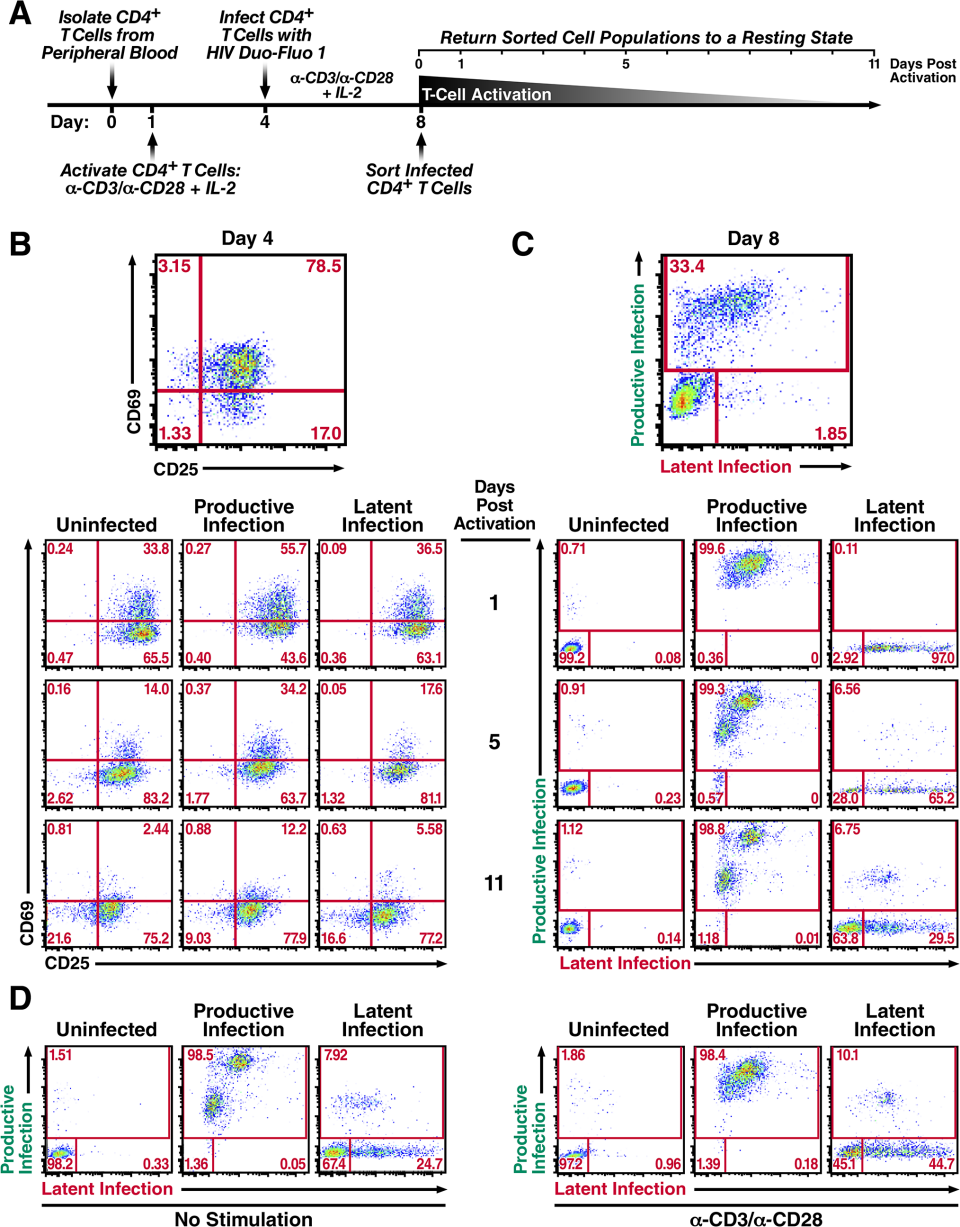
Productively infected and latently infected primary CD4^+^ T cells isolated by FACS return to a resting state. (A) Schematic of experimental procedure. Primary CD4^**+**^ T cells were isolated from uninfected donor blood and stimulated with αCD3/αCD28 activating beads in the presence of IL-2 for 72 h and then infected with HIV Duo-Fluo I virus. Productive, latent, and uninfected cell populations were isolated via FACS 4 days after infection and were allowed to return to a resting state for 11 days in the presence of IL-2. (B) Expression of activation markers CD69 and CD25 in each isolated population as they return to a resting state. (C) Infection profiles of each isolated population as they return to a resting state, as analyzed by flow cytometry. (D) Reactivation of isolated cell populations after returning to a resting state. Cells were split in half and either left unstimulated or stimulated with αCD3/αCD28 activating beads for 48 h. All data shown are from a single donor, but representative of three separate donors.

To explore another possible way latency is established, we next investigated whether productively infected activated primary CD4^+^ T cells can return to a resting state and contribute to the latent reservoir. We isolated CD4^+^ T cells from uninfected donor blood and activated them with αCD3/αCD28 activating beads in the presence of IL-2 for 3 days. At this point, we spinoculated the cells with HIV Duo-Fluo I for 2 h at 37°C (Fig 3A). After infection, cells were kept in an activated state by returning them to culture in the presence of activating beads and IL-2. Four days postinfection, CD4^+^ T cells were sorted to isolate three distinct populations: uninfected (GFP-/mCherry-), productively infected (GFP+/mCherry- & GFP+/mCherry+), and latently infected (GFP-/mCherry+) cells. After sorting, a small fraction of each population was used to measure HIV integration via Alu-gag PCR (S5 Fig), while the majority of each population was cultured with IL-2 and allowed to return to a resting state over an 11-day period.

The activation state of each cell population was monitored by the expression of the activation markers CD69 and CD25 (Fig 3B), as well as changes in cell size (S6 Fig). The activation markers CD69 and CD25 were maximally expressed at day 4, when the cells were infected with HIV Duo-Fluo I (Fig 3B). After sorting, each cell population began to lose both CD69 and CD25 expression, and the cell size of each population began to shrink (S6 Fig). However, none of the distinct cell populations fully returned to resting during the 11-day period. The uninfected cell population contained 21.6% of CD69 and CD25 double-negative cells at 11 days post-activation compared to 16.6% for the latently infected population and just 9.03% for the productively infected population. This indicates that the uninfected population is returning to a resting state more quickly than either of the infected cell populations. Additionally, the productively infected cell population contained 12.2% CD69 and CD25 double positive cells at 11 days post-activation compared to 5.58% for the latently infected cell population and just 2.44% for the uninfected cell population, indicating that the productively infected CD4^+^ T cells are returning to a resting state at a slower rate than either latently infected or uninfected CD4^+^ T cells. As such, latently infected cells are less activated than productively infected cells.

While activated cells were returning to a resting state over the 11-day period, they experienced changes in their infection profiles (productive or latent infection) (Fig 3C). A small percentage of the uninfected cell population became infected, which most likely reflects pre-integration latency [18]. More interestingly, 98.8% of the productively infected cell population continued to express GFP throughout the 11 days, with 1.18% of the cells no longer expressing GFP. Based on these data, only a small fraction of productively infected cells have returned to a resting state over the course of the 11 days, and potentially contribute to the latent reservoir.

Over the course of the 11 days, the latently infected cell population began to display two distinct phenotypes. First, over 6% of the latently infected cell population spontaneously reactivated as exhibited by their GFP expression (Fig 3C). Second, over 60% of the latently infected cells lost expression of mCherry, in addition to not expressing GFP. By analyzing the activation marker expression profile for these distinct populations (S7 Fig), we found that those cells that lost mCherry expression had higher levels of CD69/CD25 double-negative cells (24.9%) than the latently infected cells that continued to express mCherry (12.8%) and those that spontaneously reactivated (3.23%). Taken together, these data demonstrate that the majority of activated CD4^+^ T cells that become latently infected revert back to a resting state, while also silencing the EF1-α-driven mCherry expression.

After allowing the sorted cell populations to return to a resting state for 11 days, we stimulated each population with αCD3/αCD28 activating beads to reactivate any latent provirus (Fig 3D). In the uninfected cell population, stimulation produced a small amount of reactivatable provirus which, again, is likely due to pre-integration latency [18]. Interestingly, stimulation of the productively infected cell population did not lead to reactivation of the ~1% of cells that no longer expressed GFP. Lastly, stimulation of the latently infected cell population produced only a small amount of reactivatable provirus (~2%). However, we observed a large shift from latently infected cells that no longer express mCherry to latently infected cells that do express mCherry, as evidenced by a 20% increase in mCherry+ cells after αCD3/αCD28 stimulation (Fig 3D). These results indicate that latently infected cells that lose mCherry expression over time are not dying but instead returning to a resting state.

### Primary CD4^+^ T cells within *ex vivo* lymphoid tissue are biased toward latent HIV infection, but they can also support productive infection

**Fig 4:**
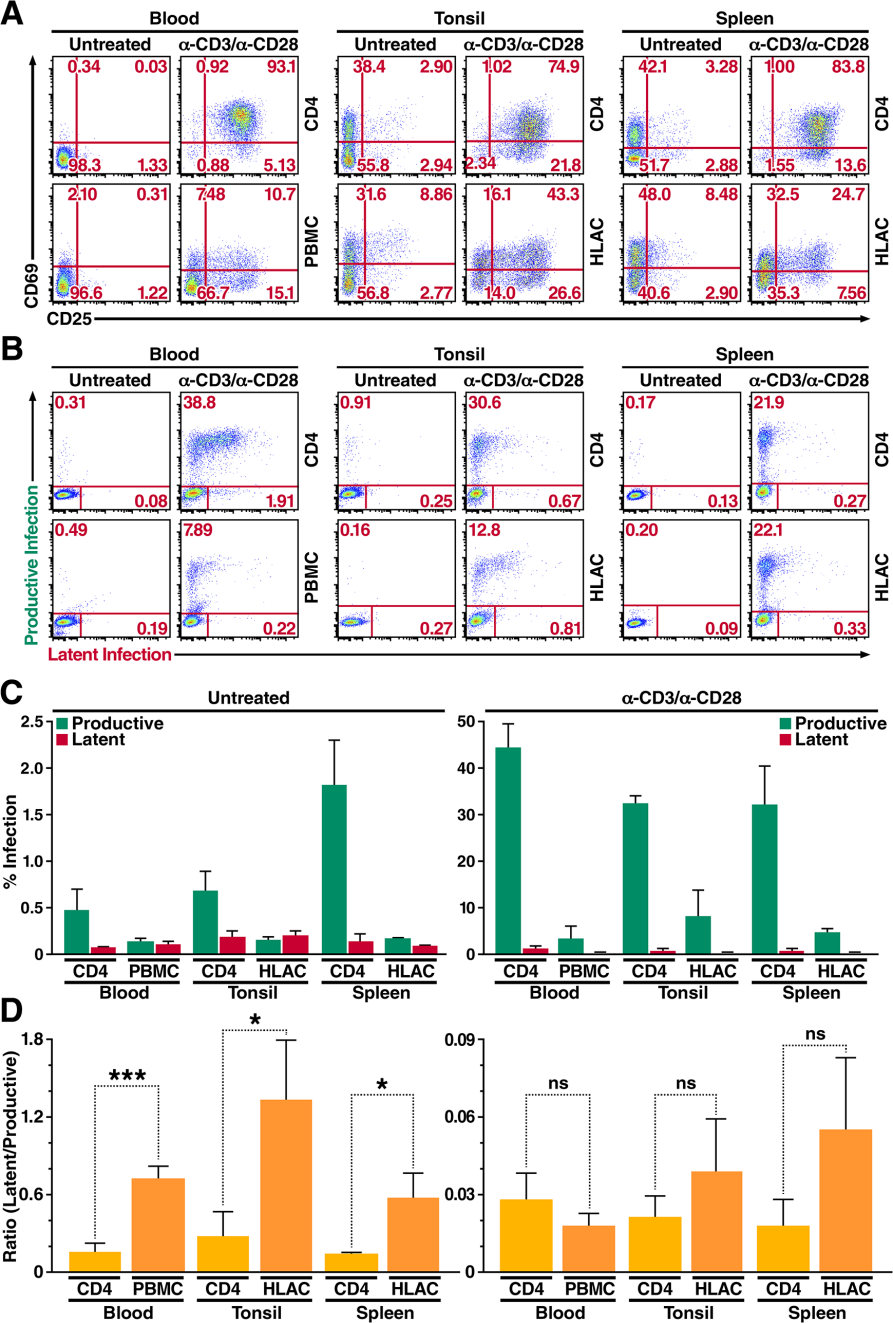
Primary CD4^+^ T cells and total lymphoid cell populations isolated from peripheral blood and tonsillar and splenic tissues are more likely to become latently infected. (A) Expression of activation markers CD69 and CD25 on CD4^**+**^ T cells and total lymphoid cell populations either left untreated or stimulated with αCD3/αCD28 activating beads for 72 h. Data shown are from a single donor, but are representative of three separate donors. (B) Infection profiles of CD4^**+**^ T cells and total lymphoid cell populations from panel A. Cells were infected with HIV Duo-Fluo I and analyzed for productive and latent infection 72 h after infection. Data shown are from a single donor, but are representative of three separate donors. (C) Quantification of latent infection and productive infection from panel B. Data represent the average of three donors. (D) Ratios of latent infection to productive infection were calculated using data from panel C. Data represent the average of three donors. *, P < 0.05; ***, P < 0.001; ns, nonsignificant.

Lastly, based on evidence that resting CD4^+^ T cells within lymphoid tissue can support HIV replication [34–39], we wanted to investigate how latency is established within *ex vivo* lymphoid tissue using our HIV Duo-Fluo I virus. We isolated CD4^+^ T cells from tonsillar and splenic tissues, as well as from peripheral blood from uninfected donors. Because lymphoid organs contain over 98% of the body’s CD4^+^ T cells, and are the primary sites of HIV replication, we also isolated total lymphoid cells from tonsillar and splenic tissues from uninfected donors in the form of human lymphoid aggregated cultures (HLACs), which closely mimic the conditions encountered by HIV, *in vivo* [36]. In addition, we isolated total peripheral blood mononuclear cells (PBMCs) from uninfected donors. CD4^+^ T cells, PBMCs, and HLACs were either left untreated or stimulated with αCD3/αCD28 activating beads in the presence of IL-2 for 3 days and then spinoculated with HIV Duo-Fluo I for 2 h at 37°C. We assessed expression of the activation markers CD69 and CD25 before and after stimulation (Fig 4A). PBMCs and CD4^+^ T cells isolated from peripheral blood expressed very little CD69 or CD25 and were thus considered resting cells. However, CD4^+^ T cells isolated from tonsillar and splenic tissues, as well as HLACs from these tissues, highly expressed the early activation marker CD69 (38% and 42%, respectively) but expressed low levels of the intermediate activation marker CD25. Thus, CD4^+^ T cells isolated from lymphoid tissue are not resting cells, but they are also not fully activated. After stimulation with αCD3/αCD28 activating beads in the presence of IL-2 for 3 days, CD4^+^ T cells from all three tissues expressed high levels of both activation markers, reflecting classic T cell activation. However, upon stimulation, CD4^+^ T cells from peripheral blood achieved higher activation levels than CD4^+^ T cells isolated from either lymphoid tissue. Lastly, expression of CD69 and CD25 among αCD3/αCD28-stimulated PBMCs and HLACs was consistently lower than in purified CD4^+^ T cells, and may reflect the size of the CD4^+^ T cell population within each culture.

Levels of productive and latent infection were analyzed by flow cytometry 72 h post-infection (Fig 4B and 4C). Untreated CD4^+^ T cells from peripheral blood, which expressed no activation markers, produced very little productive infection (0.47%). Despite expressing moderately high levels of CD69, untreated CD4^+^ T cells isolated from tonsillar tissue did not give rise to significantly higher levels of productive infection (0.68%) compared to CD4^+^ T cells from peripheral blood. However, untreated CD4^+^ T cells from splenic tissue—which expressed CD69 at levels comparable to those of CD4^+^ T cells isolated from tonsillar tissue—did show an increase in productive infection (1.8%) as compared to untreated CD4^+^ T cells from peripheral blood. Levels of latent infection in untreated CD4^+^ T cells isolated from both lymphoid tissues were at least 2-fold greater than those observed in untreated CD4^+^ T cells from peripheral blood (Fig 4C), suggesting that CD4^+^ T cells within lymphoid tissue are more likely to become latently infected.

Overall, untreated PBMCs and untreated HLACs from both lymphoid tissues displayed lower levels of productive infection than untreated CD4^+^ T cells isolated from each tissue (Fig 4C). Infection of untreated PBMCs resulted in a 3.5-fold decrease in productive infection as compared to untreated CD4^+^ T cells from the blood, while infection of untreated HLACs from tonsil resulted in a 4-fold decrease in productive infection as compared to untreated CD4^+^ T cells from the same tissue. Untreated HLACs from the spleen resulted in an 11-fold decrease in productive infection as compared to untreated CD4^+^ T cells from the same tissue. In contrast, the levels of latent infection did not change between untreated total lymphoid cells and untreated CD4^+^ T cells from each tissue (Fig 4C). In addition, despite differences in activation levels, infection of untreated HLACs from both lymphoid tissues did not result in an increase of either productive or latent infection as compared to untreated PBMCs.

When analyzing the ratio of latent infection to productive infection, we found that HIV Duo-Fluo I infection of all untreated cells from the three different tissues was at least 5-fold more likely to result in latent infection than their αCD3/αCD28-stimulated counterparts (Fig 4D). Thus, activated cells exhibit a higher propensity for productive infection, while resting cells exhibit a higher propensity for latent infection. Additionally, infecting untreated total lymphoid cell populations results in more latent infection than when infecting purified untreated CD4^+^ T cells from the same tissue, suggesting that co-culture of CD4^+^ T cells with other lymphoid cells promotes latent infection.

## Discussion

The role that T cell activation plays in establishing HIV latency within CD4^+^ T cells is still not fully understood. HIV replication is clearly most efficient in activated CD4^+^ T cells [19–22], and the largest *in vivo* latent reservoir is within memory CD4^+^ T cells [11, 12]. This evidence suggests that HIV latency is established in one of two ways: 1) Activated CD4^+^ T cells become productively infected but survive viral cytopathic effects and evade elimination by the immune system long enough for the cell to transition to a resting memory state; or 2) CD4^+^ T cells that are transitioning from an activated to a resting memory state are infected by HIV while the cellular environment can still support integration of viral cDNA but cannot support proviral transcription. However, studies have shown that both naive and memory CD4^+^ T cells contain integrated viral DNA [34], and that direct infection of resting CD4^+^ T cells in lymphoid tissue results in productive infection [40]. These findings suggest that HIV latency can also be established in another way: direct infection of resting CD4^+^ T cells. In this study, we show that all three scenarios can produce latent HIV infection. We further show that HIV latency can be established in activated CD4^+^ T cells without them first returning to a resting state. Additionally, infecting activated CD4^+^ T cells is more likely to result in productive infection, while infecting resting CD4^+^ T cells is more likely to result in latent infection. Finally, HIV latency is more likely to occur in resting lymphoid cell aggregates than in resting CD4^+^ T cells cultured alone.

Using primary CD4^+^ T cells isolated from the blood of uninfected donors, we demonstrate that infecting resting and activated CD4^+^ T cells with our HIV Duo-Fluo I virus causes both productive and latent infection in the two populations. In activated CD4^+^ T cells, HIV latency is established within the first few days of infection and does not require the cell to return to a resting state. We showed this previously [18], as did another group that developed a similar dual-reporter virus [54]. That construct uses a different LTR-independent promoter (CMV) than our EF1α promoter, and it places the LTR-driven eGFP cassette in the Gag region, while ours replaces the Nef open reading frame. Despite these differences, both dual-reporter viruses can detect latent infection events in activated CD4^+^ T cells early after the initial infection, and these latently infected cells can be reactivated by different stimuli. Additionally, we sorted these latently infected cells and showed that they still express significant amounts of both CD69 and CD25 activation markers; the cells only stop expressing these markers as they are allowed to return to a resting state. As they return to a resting state, latently infected CD4^+^ T cells also stop expressing the EF1α-driven mCherry marker, suggesting that as these cells return to resting, both promoters become silenced, perhaps by packaging into heterochromatin [55]. This means that HIV latency may be established after activated CD4^+^ T cells are initially infected, and it is these cells, potentially, that survive and return to a resting memory state, thus significantly contributing to the latent pool. How HIV latency is established in activated CD4^+^ T cells immediately after infection is still unknown, but it may arise from stochastic viral gene expression [56–59].

Our studies also suggest that activated CD4^+^ T cells that become productively infected can contribute to the latent pool as they return to a resting state. In our studies, these cells did not return to a completely resting state because so many of the cells died (S6 Fig), a likely consequence of viral cytopathic effects. However, the data clearly indicate that a small population of productively infected cells starts to return to a resting state and as they do, they lose expression of the LTR-driven GFP marker. However, when these cells were then stimulated with αCD3/αCD28, they failed to express GFP, suggesting that they could not be reactivated by CD3/CD28 stimulation, though it is possible that other reactivating agents could work. Finally, it is important to note that these productively infected CD4^+^ T cells that did eventually shut down LTR-driven GFP expression, did so in a culture dish. It remains to be seen, *in vivo*, if productively infected CD4^+^ T cells can survive long enough to return to a resting state and contribute to the latent pool, or if activated CD4^+^ T cells that become latently infected immediately after infection are the major contributors.

Finally, infecting activated CD4^+^ T cells produces more productively infected cells than latently infected cells, while infecting resting CD4^+^ T cells produces more latently infected cells. These results reflect that HIV replicates more efficiently in activated CD4^+^ T cells, but they also show that resting CD4^+^ T cells can support HIV infection, at least up to the point of viral integration.

In resting primary CD4^+^ T cells, we show that both productive and latent HIV infection can be achieved, though at levels much lower than those seen in activated CD4^+^ T cells. The infection kinetics in resting CD4^+^ T cells seem to be slower than in activated cells, since peak infection was not reached until 6 days after infection, while activated cells reached peak infection 4 days after infection (S1 Fig). These results agree with other’s findings [22, 60]. Also in agreement with previous findings, resting CD4^+^ T cells were made more permissive to HIV infection when exposed to the chemokine CCL19, which increases the ability of resting CD4^+^ T cells to support latent infection [47]. However, our data demonstrate that CCL19 also increases permissibility to productive infection, although its overall effect on resting CD4^+^ T cells increases latent infection. Interestingly, the cytokine, IL-7, which increases permissibility of resting CD4^+^ T cells to productive HIV infection, also increased both productive and latent infection in resting CD4^+^ T cells in our study. Lastly, infecting untreated resting CD4^+^ T cells with a Vpx-containing virus significantly increased productive infection but only modestly increased latent infection.

Interestingly, infecting resting CD4^+^ T cells with our HIV Duo-Fluo I virus produced a significant amount of silent infection events, in which expression of both fluorescent proteins was silenced, camouflaging latently infected cells within our uninfected population. In fact, the isolated uninfected population of resting CD4^+^ T cells contained more silently infected cells than the number of latently infected cells that were identified via the mCherry fluorescent marker after the initial infection. This was true for all untreated and treated resting CD4^+^ T cells, except CCL19-treated cells, and was highest in IL-7-treated cells and untreated resting CD4^+^ T cells infected with a Vpx-containing virus. The reasons for this are unclear. Within resting CD4^+^ T cells, viral integration occurs in regions of the host genome that are unfavorable for viral gene expression [61], and studies also suggest that latently infected cells are more likely to contain provirus in or near heterochromatin [62, 63]. Integration into such regions would be unfavorable not only for LTR-driven gene expression but also for EF1α-driven gene expression. In the presence of Vpx, SAMHD1 cannot inhibit HIV reverse transcription, allowing the virus to bypass one of the major obstacles to replication in resting CD4^+^ T cells. Therefore, integration of the viral cDNA may occur more readily in these unfavorable heterochromatic regions. Treating cells with IL-7, which signals through the JAK/STAT pathway [64], may produce a similar situation.

Lastly, previous studies have reported that resting CD4^+^ T cells can only be infected by HIV in the context of total lymphoid cell aggregates [41]. However, our results show that infecting untreated resting CD4^+^ T cells (alone) and untreated resting total lymphoid cells from peripheral blood and lymphoid tissue all produced productive and latent populations. Although, we did find that latent infection is more likely to occur in total resting lymphoid cell aggregates than in resting CD4^+^ T cells alone. The reasons for this are still unclear, but recent studies have shown that co-culture of resting CD4^+^ T cells with myeloid dendritic cells [65], or co-culture of resting CD4^+^ T cells with endothelial cells [66], enhances HIV latency, further proving that the lymphoid environment plays an important role in how HIV latency is established within resting CD4^+^ T cells.

Overall, our studies show that HIV infection can occur in both resting and activated CD4^+^ T cells, such that infection of resting cells more often results in latent infection and infection of activated cells more often results in productive infection. Based on our data, we now have a better understanding of the contribution that each infected cell type makes to the latent reservoir. Our study underscores why we must consider both resting and activated CD4^+^ T cells when investigating how HIV latency occurs.

## Materials and Methods

### Virus production

Pseudotyped HIV Duo-Fluo I viral stocks were generated by co-transfecting (using the standard calcium phosphate transfection method) HEK293T cells with a plasmid encoding HIV Duo-Fluo I and a plasmid encoding HIV-1 dual-tropic envelope (pSVIII-92HT593.1). We generated a Vpx-containing HIV Duo-Fluo I pseudotyped virus by co-transfecting HEK293T cells with the HIV Duo-Fluo I plasmid, the pSVIII-92HT593.1 plasmid, and a plasmid encoding a Vpr-Vpx fusion protein (pSIV3+, generously donated by Warner Greene). Supernatants were collected after 72 h and filtered through a 0.45 μM membrane to clear cell debris, and were then concentrated by ultracentrifugation (76,755 x g) for 2 h at 4°C. Concentrated virions were resuspended in complete media and stored at -80°C. Virus concentration was estimated by p24 titration (HIV-1 alliance p24 ELISA kit, Perkin-Elmer).

### Primary cell isolation and cell culture

Primary CD4^+^ T cells and peripheral blood mononuclear cells (PBMCs) were purified from healthy donor blood (Blood Centers of the Pacific, San Francisco, CA, USA and Stanford Blood Center). CD4^+^ T cells were isolated by negative selection using the RosetteSep Human CD4^+^ T Cell Enrichment Cocktail (StemCell Technologies). PBMCs were purified by Histopaque-1077 density gradient. Purified resting CD4^+^ T cells and PBMCs from peripheral blood were cultured in RPMI 1640 medium supplemented with 10% fetal bovine serum (FBS), L-glutamine (2 mM), penicillin (50 U/ml), and streptomycin (50 mg/ml). Human lymphoid aggregate cultures (HLACs) were purified using tonsillar or splenic tissue from uninfected donors (Cooperative Human Tissue Network) as previously described [67]. CD4^+^ T cells were isolated from HLACs by negative selection using the EasySep Human CD4^+^ T Cell Enrichment Kit (StemCell Technologies). HLACs and CD4^+^ T cells isolated from splenic and tonsillar tissues were cultured in RPMI 1640 supplemented with 20% heat-inactivated FBS, 100 mg/ml gentamicin, 200 mg/ml ampicillin, 1 mM sodium pyruvate, 1% nonessential amino acids (Mediatech, Manassas, VA, USA), 2 mM L-glutamine, and 1% fungizone (Invitrogen, Indianapolis, IN, USA)

### Cell treatment and infection

Purified resting CD4^+^ T cells were either left untreated or treated for 3 days with 20 ng/ml IL-7 (R&D Systems) or 100 μM CCL19 (R&D Systems). Purified CD4^+^ T cells isolated from peripheral blood and tonsillar and splenic tissues, as well as PBMCs and HLACs, were stimulated with αCD3/αCD28 activating beads (Life Technologies) at a concentration of 1 bead/cell in the presence of 30 U/ml IL-2 (PeproTech) for 3 days. All cells were spinoculated with either HIV Duo-Fluo I alone or Vpx-containing HIV Duo-Fluo I at a concentration of 100 ng of p24 per 1 × 10^6^ cells for 2 h at 1,200 × *g* at 37°C. After spinoculation, all cells were returned to culture in the presence of 30 U/ml IL-2, except for CD4^+^ T cells pre-stimulated with αCD3/αCD28 activating beads, which were placed back in culture with the αCD3/αCD28 activating beads and 30 U/ml IL-2.

### Flow cytometry and cell sorting

Uninfected cells were stained in fluorescence-activated cell sorting (FACS) buffer (phosphate buffered saline supplemented with 2 mM EDTA and 2% FBS) with αCD69-PE and αCD25-APC (eBioscience) and fixed in 1% paraformaldehyde. Infected cells were stained in FACS buffer with αCD69-V450 and αCD25-APC/Cy7 (BD Biosciences) and fixed in 1% paraformaldehyde. Data were collected on a FACS Caliber and a FACS LSRII (BD Biosciences), and analyses were performed with FlowJo software (TreeStar). Untreated and treated CD4^+^ T cells from Figs 1F and S4 were sorted with a FACS AriaII (BD Biosciences) based on their GFP and mCherry fluorescence at 6 days post-infection, and they were placed back in culture with or without 30 μM Raltegravir (National AIDS Reagent Program). CD4^+^ T cells stimulated with αCD3/αCD28 activating beads in the presence of 30 U/ml IL-2 from Fig 3 were sorted based on their GFP and mCherry fluorescence at 4 days post-infection.

### SAMHD1 protein analysis

Untreated resting primary CD4^+^ T cells infected with either HIV-Duo-Fluo I alone or Vpx-containing HIV Duo-Fluo I were lysed 6 days post-infection in radioimmunoprecipitation assay buffer (150 mm NaCl, 1% Nonidet P-40 (vol/vol), 0.5% AB-deoxycholate (vol/vol), 0.1% sodium dodecyl sulfate (SDS) (vol/vol), 50 mm Tris-HCl (pH 8), 1 mm DTT, and EDTA-free Protease Inhibitor (Calbiochem). Cell lysates were used for SDS-polyacrylamide gel electrophoresis (SDS-PAGE) immunoblotting analysis. The primary antibodies used were rabbit polyclonal anti-SAMHD1 (Sigma-Aldrich, Cat# SAB2102077) and monoclonal anti-β-actin (A5316, Sigma-Aldrich).

### HIV integration

DNA was prepared after cell sorting of uninfected, productively infected and latently infected cell populations using the DNeasy Kit (Qiagen). Real-time PCR was used to detect total HIV DNA, β-globin, and integrated HIV DNA as previously described [68].

## Supporting Information

S1 Fig Resting primary CD4^+^ T cells reach peak infection levels with HIV Duo-Fluo I 6 days post infection. Infection profiles of untreated or stimulated primary CD4^+^ T cells over the course of 6 days after infection. Untreated resting CD4^+^ T cells were infected with either HIV Duo-Fluo I virus alone or the Vpx-containing HIV Duo-Fluo I virus. Stimulated cells were infected with HIV Duo-Fluo I alone. (A) Latent infection (mCherry+) and (B) productive infection (GFP+ and GFP/mCherry double-positive) were analyzed by flow cytometry every 24 hrs following infection.
(PDF)

S2 Fig Env-deficient HIV Duo-Fluo I does not contain any replication-competent virus. Primary CD4^+^ T cells were either mock infected or infected with either a replication-competent HIV-GFP (NLENG1, David N. Levy, University of Alabama, Birmingham) or one of three distinct batches of env-deficient HIV Duo-Fluo I (primary infection). Four days post-infection, supernatant was collected from the cultures, cleared of cell debris via centrifugation, and applied to freshly activated primary CD4^+^ T cells (secondary infection). Secondary infection was monitored for 12 days following infection. (A) Infection profiles for primary and secondary infections of activated primary CD4^+^ T cells. Data shown are from a single donor but are representative of three separate donors. (B) Quantified values of latent infection and productive infection from primary infections in panel A. (C) Quantified values of latent infection and productive infection from secondary infections in panel A. Data from panels B and C represent the average of three donors.
(PDF)

S3 Fig Infection of primary CD4^+^ T cells with HIV Duo-Fluo I containing Vpx leads to SAMHD1 degradation and has no effect on T cell activation. (A) Protein expression levels of SAMHD1 in resting primary CD4^+^ T cells infected with either HIV Duo-Fluo I alone or HIV Duo-Fluo I containing Vpx at 6 days after infection. (B) Expression of activation markers CD69 and CD25 in untreated resting primary CD4^+^ T cells infected with either HIV Duo-Fluo I alone or HIV Duo-Fluo I containing Vpx at 6 days after infection. Data shown are from a single donor, but are representative of three separate donors.
(PDF)

S4 Fig Untreated and treated resting primary CD4^+^ T cells contain reactivatable pre-integration and post-integration latency. Infection profiles for reactivation of pre-integration latent virus and post-integration provirus used to quantify data in Fig 1F. Data shown are from a single donor, but are representative of three separate donors.
(TIF)

S5 Fig HIV Duo-Fluo I integration events are found within the sorted productive infection and latent infection populations, but not in the uninfected population. Measure of integration events/cell within the sorted populations of activated primary CD4^+^ T cells. Data represents the average of three donors.
(PDF)

S6 Fig Cell-size changes of productive, latent, and uninfected cell populations as they return to a resting state. Productive (green), latent (red) and uninfected (black) primary CD4^+^ T cell populations were analyzed for cell-size changes via the use of the forward scatter parameter (FSC-A) 1, 5 and 11 days post activation, and compared to the cell-size of the untreated, resting population, and the αCD3/αCD28-treated population at day 4 (Fig 3B). Data shown are from a single donor, but are representative of three separate donors.
(PDF)

S7 Fig Latently infected primary CD4^+^ T cells that lose expression of their fluorescent markers are more likely to exhibit a resting phenotype. (A) Expression of activation markers CD69 and CD25 within GFP/mCherry double-negative (1), mCherry single-positive (2) and GFP/mCherry double-positive (3) cells from latently infected primary CD4^+^ T cells at 11 days after activation (Fig 3C).
(PDF)
