# Supplementary figures and images for: HIV Latency Is Established Directly and Early in Both Resting and Activated Primary CD4 T Cells
Source: PLoS Pathog. 2015 Jun 11;11(6):e1004955. doi: 10.1371/journal.ppat.1004955 (PMC4466167; doi:10.1371/journal.ppat.1004955)

**A**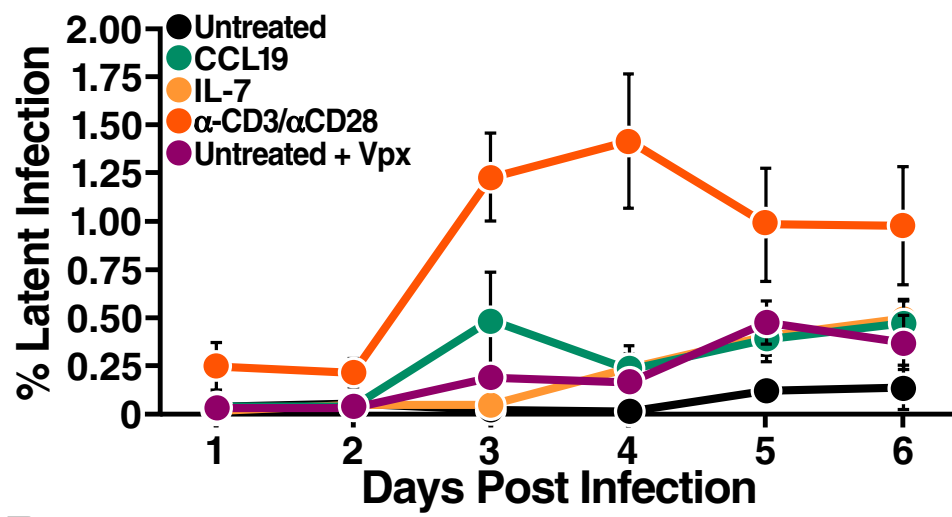**B**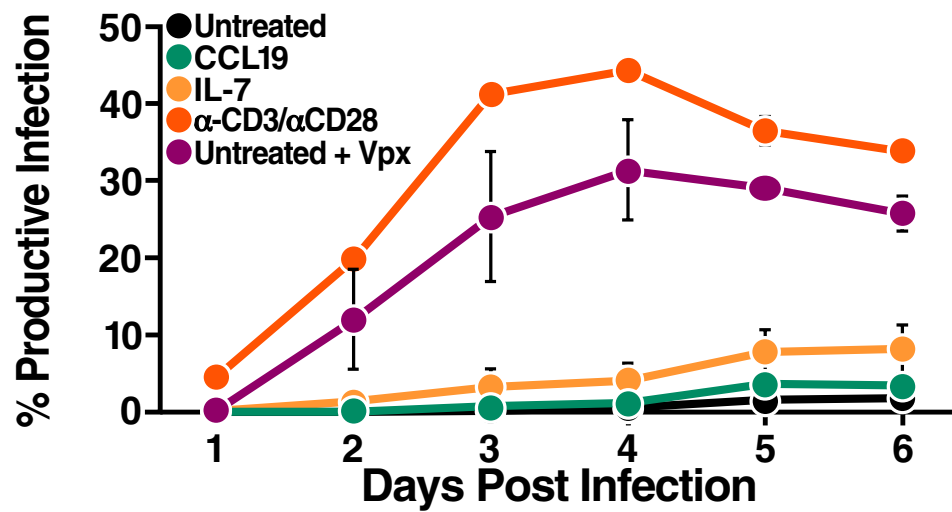

Supplement: S1 Fig — Infection profiles of untreated or stimulated primary CD4+ T cells over the course of 6 days after infection. Untreated resting CD4+ T cells were infected with either HIV Duo-Fluo I virus alone or the Vpx-containing HIV Duo-Fluo I virus. Stimulated cells were infected with HIV Duo-Fluo I alone. (A) Latent infection (mCherry+) and (B) productive infection (GFP+ and GFP/mCherry double-positive) were analyzed by flow cytometry every 24 hrs following infection. (PDF) [file ppat.1004955.s001.pdf]

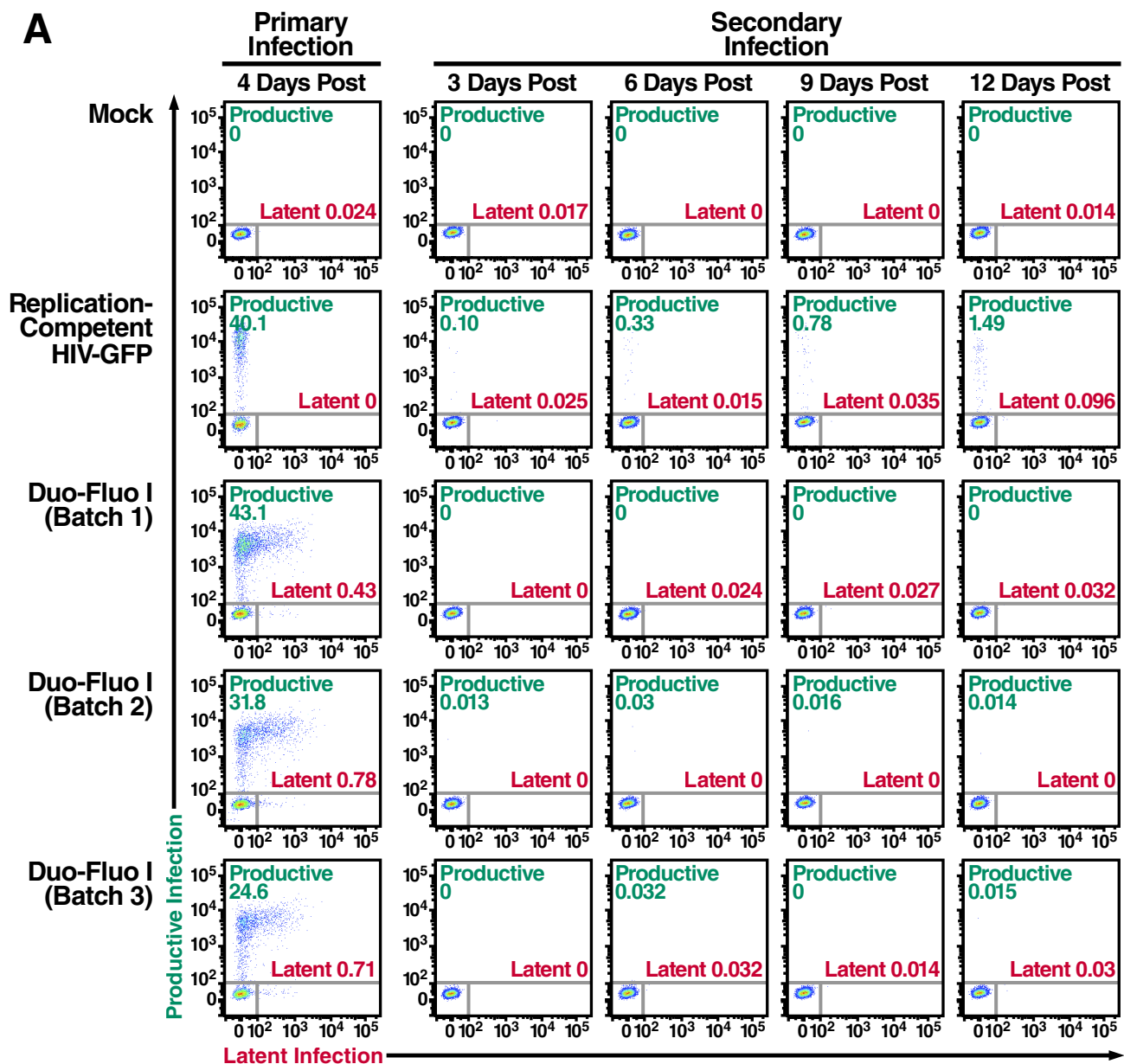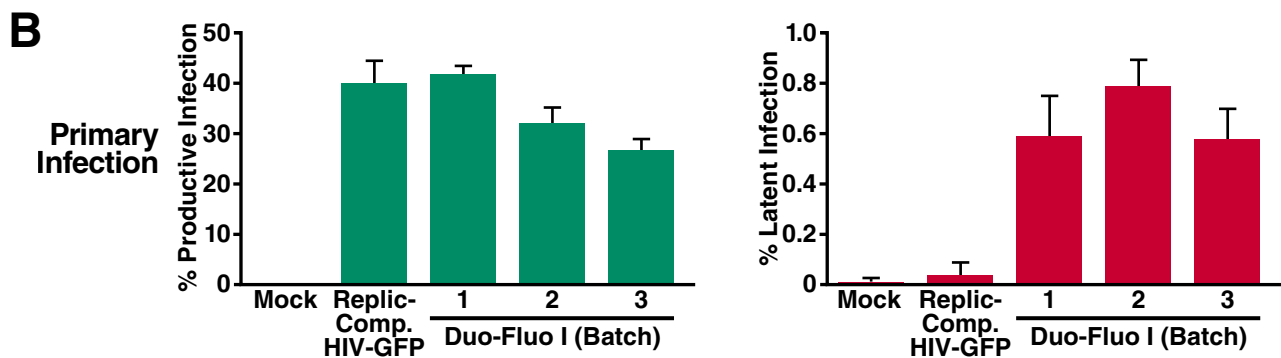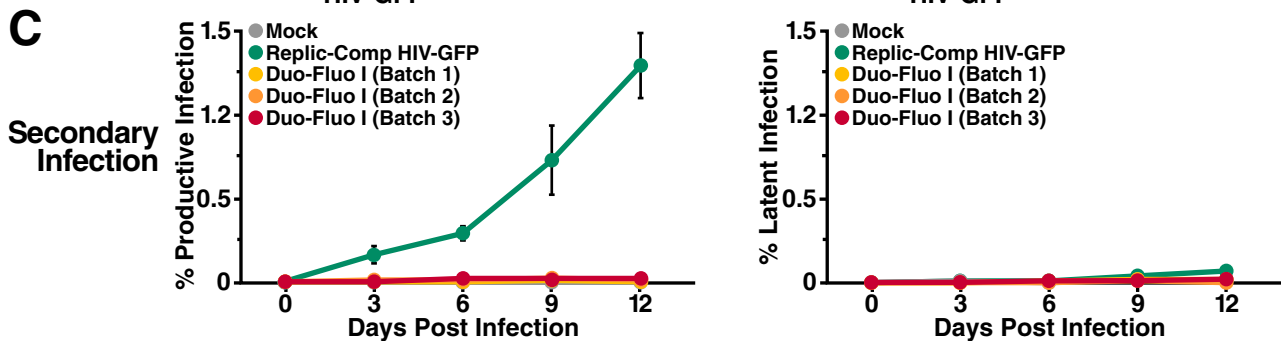

Supplement: S2 Fig — Primary CD4+ T cells were either mock infected or infected with either a replication-competent HIV-GFP (NLENG1, David N. Levy, University of Alabama, Birmingham) or one of three distinct batches of env-deficient HIV Duo-Fluo I (primary infection). Four days post-infection, supernatant was collected from the cultures, cleared of cell debris via centrifugation, and applied to freshly activated primary CD4+ T cells (secondary infection). Secondary infection was monitored for 12 days following infection. (A) Infection profiles for primary and secondary infections of activated primary CD4+ T cells. Data shown are from a single donor but are representative of three separate donors. (B) Quantified values of latent infection and productive infection from primary infections in panel A. (C) Quantified values of latent infection and productive infection from secondary infections in panel A. Data from panels B and C represent the average of three donors. (PDF) [file ppat.1004955.s002.pdf]

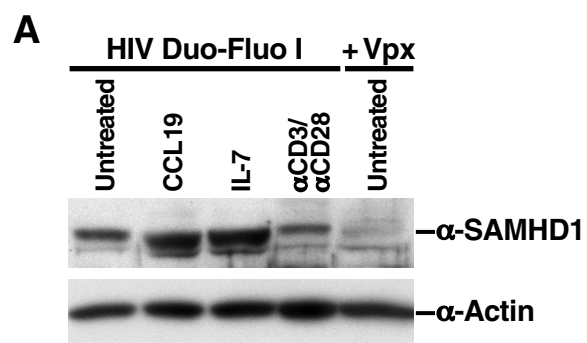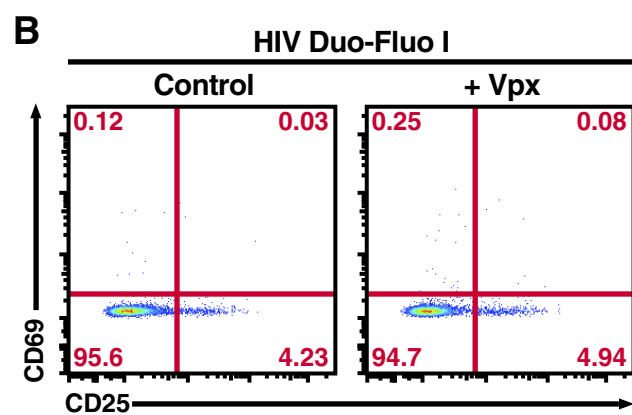

Supplement: S3 Fig — (A) Protein expression levels of SAMHD1 in resting primary CD4+ T cells infected with either HIV Duo-Fluo I alone or HIV Duo-Fluo I containing Vpx at 6 days after infection. (B) Expression of activation markers CD69 and CD25 in untreated resting primary CD4+ T cells infected with either HIV Duo-Fluo I alone or HIV Duo-Fluo I containing Vpx at 6 days after infection. Data shown are from a single donor, but are representative of three separate donors. (PDF) [file ppat.1004955.s003.pdf]

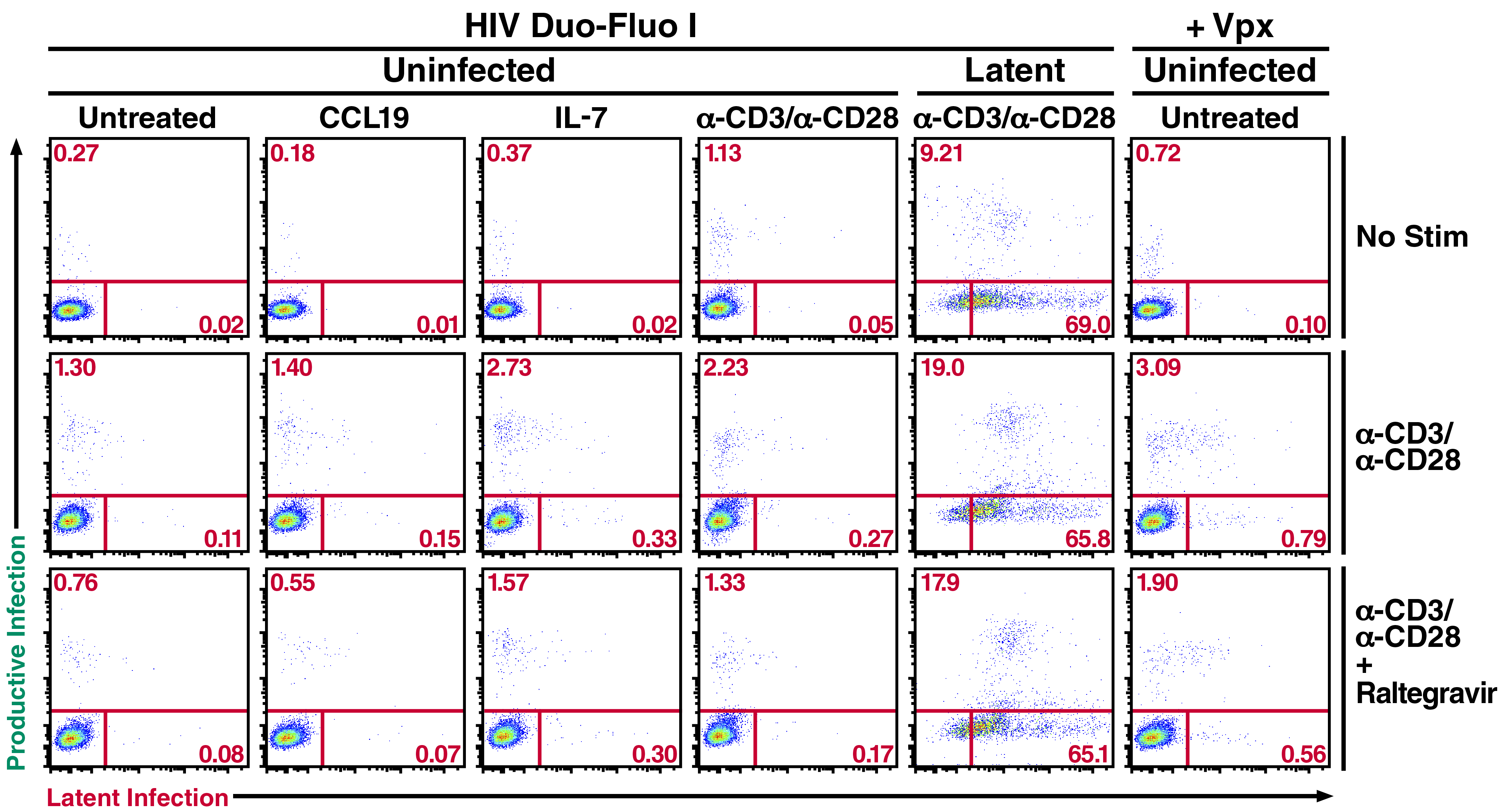

Supplement: S4 Fig — Infection profiles for reactivation of pre-integration latent virus and post-integration provirus used to quantify data in Fig 1F. Data shown are from a single donor, but are representative of three separate donors. (TIF) [file ppat.1004955.s004.tif]

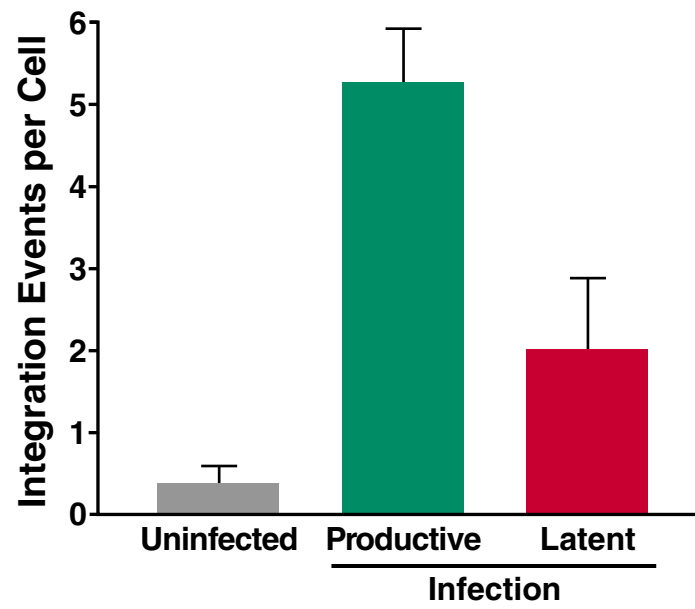

Supplement: S5 Fig — Measure of integration events/cell within the sorted populations of activated primary CD4+ T cells. Data represents the average of three donors. (PDF) [file ppat.1004955.s005.pdf]

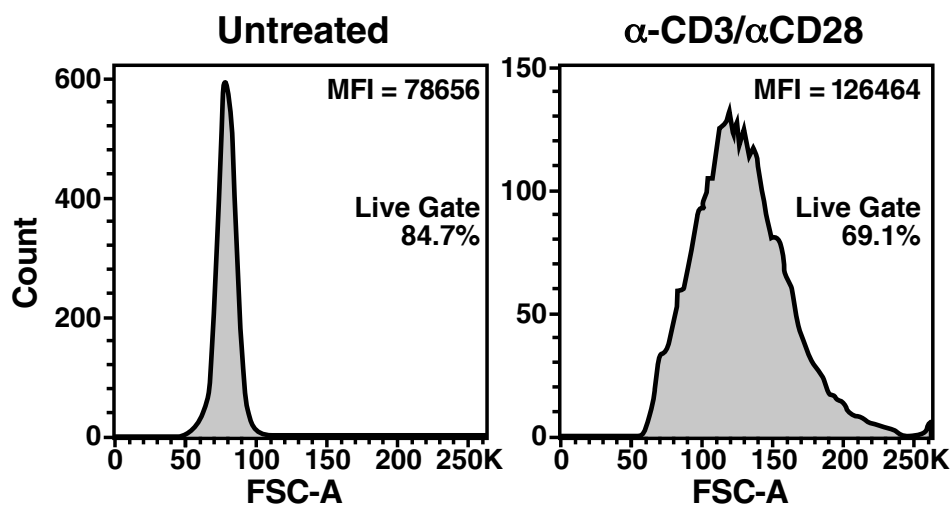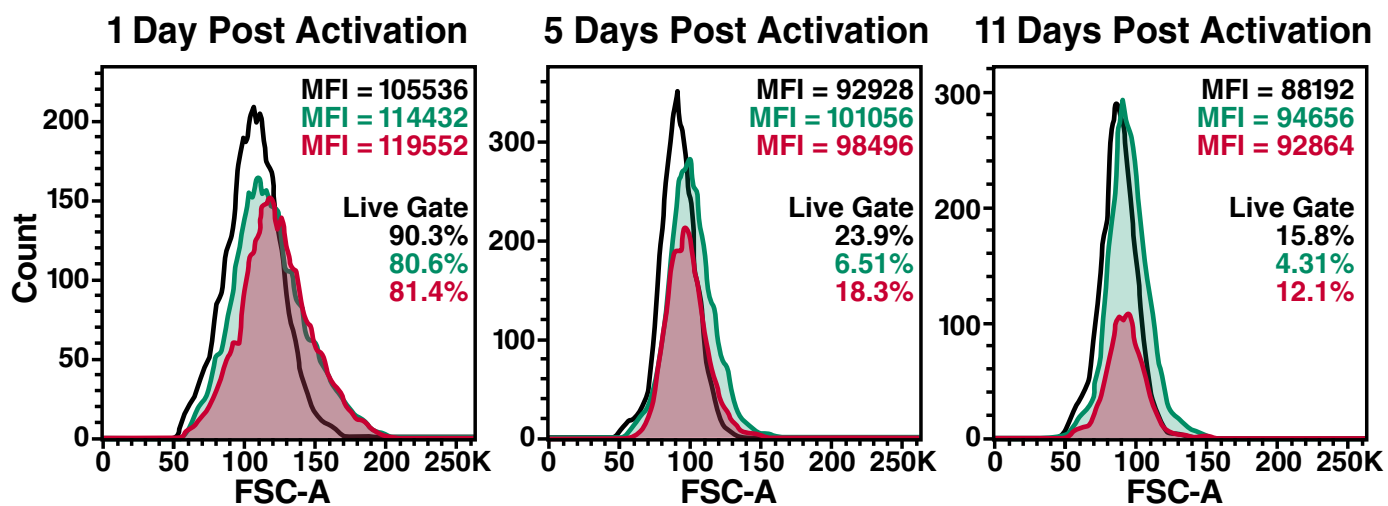

Supplement: S6 Fig — Productive (green), latent (red) and uninfected (black) primary CD4+ T cell populations were analyzed for cell-size changes via the use of the forward scatter parameter (FSC-A) 1, 5 and 11 days post activation, and compared to the cell-size of the untreated, resting population, and the αCD3/αCD28-treated population at day 4 (Fig 3B). Data shown are from a single donor, but are representative of three separate donors. (PDF) [file ppat.1004955.s006.pdf]

Latent Infection  
11 Days Post Activation

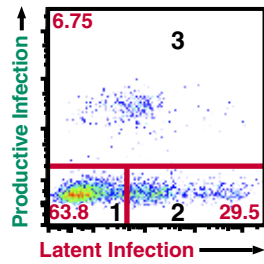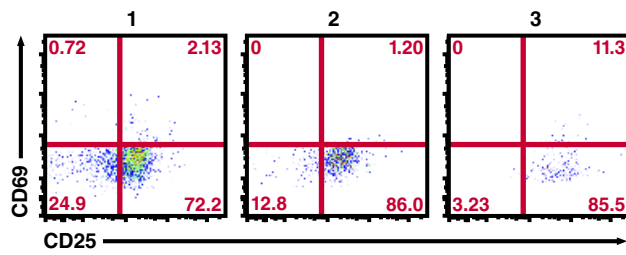

Supplement: S7 Fig — (A) Expression of activation markers CD69 and CD25 within GFP/mCherry double-negative (1), mCherry single-positive (2) and GFP/mCherry double-positive (3) cells from latently infected primary CD4+ T cells at 11 days after activation (Fig 3C). (PDF) [file ppat.1004955.s007.pdf]
